# Prevalence and Associated Factors of High-Risk Human Papillomavirus Infections among Human Immunodeficiency Virus-Infected Women in Lagos, Nigeria

**DOI:** 10.21203/rs.3.rs-4645178/v1

**Published:** 2024-07-19

**Authors:** Kehinde S. OKUNADE, Kabir B. BADMOS, Austin OKORO, Nicholas A. AWOLOLA, Francisca O. NWAOKORIE, Hameed ADELABU, Iyabo Y. ADEMUYIWA, Temitope V. ADEKANYE, Packson O. AKHENAMEN, Elizabeth ODOH, Chinelo OKOYE, Alani S. AKANMU, Adekunbiola A. BANJO, Rose I. ANORLU, Jonathan S. BEREK

**Affiliations:** University of Lagos; University of Lagos; Lagos University Teaching Hospital; University of Lagos; College of Medicine of the University of Lagos; University of Lagos; University of Lagos; Lagos University Teaching Hospital; Lagos University Teaching Hospital; Lagos University Teaching Hospital; Lagos University Teaching Hospital; University of Lagos; University of Lagos; University of Lagos; Stanford University School of Medicine

**Keywords:** Cervical cancer, HPV genotypes, Lagos, Multiple HPV infections, Screenings

## Abstract

**Background:**

Given the synergistic relationship between human immunodeficiency virus (HIV) and human papillomavirus (HPV) infections, knowledge of the genotypic prevalence and associated factors of high-risk HPV (HR-HPV) among HIV-infected women is crucial for developing targeted interventions such as appropriate screening tests and effective genotype-specific vaccination.

**Objectives:**

We determined the prevalence of any HR-HPV and multiple HR-HPV infections and identified associated factors among a cohort of women living with HIV infections (WLHIV) in Lagos, Nigeria.

**Methods:**

This descriptive cross-sectional study analysed the data of 516 WLHIV who underwent cervical cancer screening as part of the *COMPASS-DUST study* at the HIV treatment centre of Lagos University Teaching Hospital from July 2023 to March 2024. Multivariable binary logistic regression models were performed to explore factors associated with HR-HPV and multiple HR-HPV infections.

**Results:**

Among the 516 WLHIV enrolled (mean age, 46.5±7.3 years), the overall HR-HPV prevalence was 13.4% (95% CI, 10.6–16.6), disaggregated as 3.3% for HPV16/18 (95% CI, 1.9–5.2) and 11.6% for *other* HR-HPV genotypes (95% CI, 9.0–14.7). Nineteen women (3.7%; 95% CI, 2.2–5.7)had multiple HR-HPV genotype infections. Having a recent serum CD4+ cell count ≤560 cells/μL (adjusted OR 3.32; 95% CI 1.06–10.38) and HPV 16/18 genotype infections (adjusted OR 38.98; 95% CI 11.93–127.37) were independently associated with an increased risk of multiple HR-HPV infections.

**Conclusion:**

The findings of this study provide valuable insights into the epidemiology of HR-HPV infections and highlight the need for tailored interventions and continuous monitoring. By addressing these challenges through targeted screening, effective ART management, and vaccination programs, we can improve health outcomes and reduce the burden of cervical cancer in this vulnerable population.

## Introduction

Human papillomavirus (HPV) infection is a major global health concern, particularly due to its established role in the development of invasive cervical cancer [1]. Cervical cancer is a leading cause of cancer-related morbidity and mortality among women worldwide [2], with a disproportionately higher burden in low- and middle-income countries, including Nigeria, where screening and vaccination programs are less accessible [3].

Increasing evidence revealed a higher risk and poorer prognosis of cervical cancer among women with human immunodeficiency virus (HIV) compared to the general population. HIV infection exacerbates the risk of acquiring high-risk HPV (HR-HPV) infections due to the immunocompromised state it induces [4]. Therefore, due to impaired immune function, there is an increased susceptibility to persistent HR-HPV infection due to ineffective viral clearance in HIV-infected individuals [5–8] that leads to the development of cervical intraepithelial neoplasia which can progress to invasive cervical cancer, if left untreated [4, 9, 10].

Given this synergistic relationship between HIV and HPV infections, knowledge of the genotypic prevalence and associated factors of HR-HPV among HIV-infected women is crucial for developing targeted interventions such as effective genotype-specific vaccination and appropriate cervical cancer screening tests. Nigeria, the most populous country in Africa, faces significant public health challenges related to both HIV and cervical cancer [6, 11]. Lagos, the most populous state and commercial capital of Nigeria presents a microcosm of these challenges, with a high burden of HIV infection and limited resources for comprehensive cervical cancer screening and prevention [10, 12]. Despite the significant risk of HR-HPV infection, data on its prevalence among women living with HIV (WLHIV) in Lagos is sparse [12, 13], thus limiting the ability to effectively design interventions to address this dual burden risk factor of cervical cancer. This study, therefore, aimed to fill this knowledge gap by determining the prevalence of any HR-HPV and multiple HR-HPV infections and identifying associated factors among WLHIV in Lagos, Nigeria. By elucidating these factors, we hope to inform public health strategies and clinical practices that can mitigate the impact of HR-HPV and improve health outcomes for this vulnerable population.

## Participants and Methods

### Study design and settings

This is a descriptive cross-sectional analysis of the data of WLHIV who underwent cervical cancer screenings as part of the baseline assessments in the “*COMPASS-DUST*” study. “*COMPASS-DUST*” is a two-phase study currently ongoing in the HIV treatment centre of the Lagos University Teaching Hospital (LUTH). Details of the study design and procedure are published elsewhere [14]. LUTH, the leading public health institution in Lagos State, Southwest Nigeria, serves a population of over 20 million. It primarily functions as a referral centre for various government and private hospitals within Lagos and its neighbouring states. Each month, the LUTH HIV treatment clinic delivers comprehensive care to approximately 9,000 individuals living with HIV in Lagos and the surrounding states of Southwest Nigeria. The clinic also offers integrated reproductive health care to sexually active women with HIV infection such as routine cervical cancer screening with either a Pap smear, HPV DNA testing or visual inspection with acetic acid (VIA) [10].

### Eligibility criteria

We included and analyzed the data of n = 516 sexually active WLHIV aged 25–65 years who had their HR-HPV infection status recorded after undergoing pelvic examination and cervical liquid-based cytology (LBC) sample collection for cervical cancer screenings including Pap smear, p16/Ki-67 dual staining and HPV testing, at enrolment in the primary study [15] between July 2023 to March 2024. An HPV testing was conducted by a study pathologist/technician, utilizing HPV DNA target amplification by Polymerase Chain Reaction followed by nucleic acid hybridization to detect 14 high-risk HPV genotypes (types 16, 18, 31, 33, 35, 39, 45, 51, 52, 56, 58, 59, 66, and 68) in the LBC samples. Excluded from the study were women with suspicious cervical lesions; those with ongoing or recent pregnancy, those with previous HPV vaccination, those with a prior history of cervical cancer or who have had therapy for benign or malignant cervical lesions, and those who have had hysterectomy.

### Extracted variables of interest

Variables extracted for analyses in the dataset included the participant’s age in years, number of previous childbirths and vaginal births, body mass index (BMI) in kg/m^2^, marital, educational and employment status, CD4 + cell count and viral load, age at menarche, coitarche and first pregnancy, menstrual status, use of oral hormonal contraceptive, number of lifetime sex partners, previous sexually transmitted infection (STI) treatment, and history of consumption of alcoholic beverages. BMI was calculated as the participant’s weight in kilograms divided by the square of height in meters [16].

### Operational definitions of study outcomes

We assessed four study outcomes: the prevalence of any HR-HPV and multiple HR-HPV infections defined as the proportion of study participants with at least one HR-HPV genotype infection and more than one HR-HPV genotype infections, respectively; and associated factors of HR-HPV and multiple HR-HPV infections, which are variables collected at baseline in the *COMPASS-DUST* study [15] that were significantly associated with HR-HPV and multiple HR-HPV infections, respectively.

### Sample size estimation

Using Fisher’s formula [17], we estimated a post-hoc sample size of n = 322 to assess the prevalence of HR-HPV infections and multiple HR-HPV infections in WLHIV based on a type I error rate of 5% at a 95% confidence level of 1.96 and a derived proportion of 24.5% for HR-HPV and 8.2% for multiple HR-HPV infections from an earlier study by Ezechi et al. [12], assuming a missing outcome data or data recording error rate of 5%. We, therefore, included the 516 WLHIV who had their HPV infection status recorded in the primary study [14] in our data analyses.

### Statistical analysis

Data analyses were performed using IBM SPSS Statistics for Windows, Version 29.0 (IBM Corporation, Armonk, NY, USA). Continuous variables were tested for normality using the Kolmogorov–Smirnov test with Lilliefors’ significance correction and descriptive statistics were then computed for the participants’ baseline characteristics. Categorical variables were expressed as frequencies and percentages, whereas continuous variables were displayed as mean (± standard deviation). We estimated prevalence at 95% confidence intervals using the Clopper-Pearson exact test. Bivariable and multivariable binary logistic regression models were developed to identify variables associated with any HR-HPV and multiple HR-HPV infections. Age and other variables related to the study outcomes (*P* < 0.20) in the bivariable analyses were then included in the pool of variables for the multivariable analyses using the backward stepwise approach. An Akaike’s Information Criterion (AIC) was calculated continuously, and the final model step with the lowest AIC was chosen as the best-fit model. Associations in the final model were considered significant if *P* < 0.05.

## Results

A total of n=516 HIV-infected women enrolled at baseline in the primary study [15] were included in the data analyses. Out of these enrolled women (mean age, 46.5±7.3 years), n=69 (13.4%; 95% CI, 10.6–16.6) had any HR-HPV infections disaggregated as 3.3% for HPV16/18 (95% CI, 1.9–5.2) and 11.6% for other HR-HPV genotypes (95% CI, 9.0–14.7). Nineteen women (3.7%; 95% CI, 2.2–5.7) had multiple HR-HPV genotype infections. The five most common HR-HPV genotypes were HPV 16 (20.0%), HPV 52 (17.2%), HPV 31 (12.8%), HPV 58 (11.4%), and HPV 51 (10.0%). The sociodemographic and clinical characteristics of the enrolled women are presented in Table 1.

In the bivariable analyses, variables significantly associated with HR-HPV infections based on *P*<0.20 were recent CD4+ cell count ≤560 cells/μL, first pregnancy before age 23 years, nulliparity, being economically unengaged, and having previous treatment for sexually transmitted infections. However, following a multivariable analysis, none of these variables were independently associated with HR-HPV infections in WLHIV [Table 2].

As shown in Table 3, participants aged ≥47 years, with recent serum CD4+ cell count ≤560 cells/μL, alcoholic beverage consumption, and having HPV 16/18 genotype infections were associated with multiple HR-HPV infections in the bivariable analyses. In the final multivariable analysis, having a recent serum CD4+ cell count ≤560 cells/μL (adjusted OR 3.32; 95% CI 1.06–10.38) and HPV 16/18 genotype infections (adjusted OR 38.98; 95% CI 11.93–127.37) were independently associated with multiple HR-HPV infections [Table 3].

**Figure 1:**
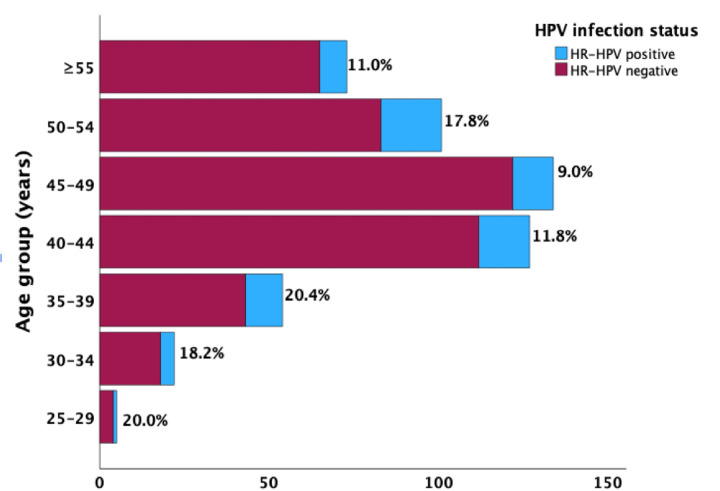
Age-specific prevalence of HR-HPV infections in enrolled HIV-infected women.

The age-specific prevalence rates of HR-HPV infections in WLHIV are shown in Figure 1. There was a trend towards a progressive decline in HR-HPV prevalence with age, with a bimodal peak prevalence seen in women aged 25–39 years (20.0–20.4%) and those in the 50–54 years age group (17.8%). The nadir prevalence was recorded in women aged 45–49 years (9.0%).

## Discussion

The findings from this descriptive cross-sectional study of HIV-infected women enrolled in the *COMPASS-DUST* study [14] provide significant insights into the prevalence and risk factors associated with high-risk human papillomavirus (HR-HPV) infections in this vulnerable population. Our study reveals that one in seven of the participants have HR-HPV infections at baseline while the prevalence of multiple HR-HPV infections was considerably lower, with only one in 27 women affected. None of the baseline participants’ factors was associated with HR-HPV infections. However, having a serum CD4+ cell count below 560 cells/μL and a co-infection with either HPV 16 or 18 genotype were significantly associated with the detection of multiple HR-HPV infections.

The prevalence of HR-HPV infections (13.4%) recorded in our study aligns with existing literature across the world, suggesting that HIV-infected individuals have a high burden of HPV infections [8]. This figure is, however, lower than the prevalence of 24.5% reported by Ezechi et al. in 2014 [12] among a predominantly similar population of women in another clinical setting in Lagos, Nigeria, and even much lower than the 42.6% recorded in a recently published study by Traore et al. [18] in Bamako, Mali, and the reported pooled estimates of 64.0% by Menon et al. in 2016 [19] and 51.0% by Bogale et al. in 2020 [20] in two systematic reviews and meta-analyses among HIV infected women in Kenya and developing countries respectively. The higher prevalence rates in these studies may be influenced by regional variations in HPV epidemiology, variations in the diagnostic methods or sensitivity of HR-HPV detection techniques, temporal differences in the study periods reflecting changes in public health interventions or HIV care, and potential variations in implementation, adherence and efficacy of antiretroviral therapy (ART) across different settings. Notably, our study enrolled only sexually active women on ART with well-controlled HIV who are otherwise healthy, hence, the relatively lower HPV prevalence we recorded.

Our study revealed that 3.7% of the enrolled WLHIV had multiple HR-HPV genotype infections. This prevalence is notably close to the 8.2% reported in a 2014 study among HIV-positive women in a similar geographical setting in Lagos, Nigeria [12]. This is, however, at substantial variance from the broader Sub-Saharan Africa regional estimates of 22.6% prevalence reported in a 20-year systematic review of WLHIV [21]. These variations in study findings may be due to regional variations in HPV epidemiology, temporal differences in the study periods reflecting changes in public health interventions or HIV care, and the population characteristics as reflected in our study where only ART adherent and healthy HIV-infected women with well-controlled infections were enrolled.

Similar to the finding by Bogale et al. [20] in 2020, HPV 16 was the most prevalent HPV genotype (20.0%) in our study as reported in most of the existing literature [1,12]. This finding was, however, different from a previous review which reported HPV 52 as the most prevalent genotype (26.0%) among HIV-infected women [19]. HPV 52 (17.2%) is the second predominant HPV genotype observed in this current study. Our findings also underscore the predominant presence of HR-HPV types 16, 31, 51, 52, and 58, among which only HPV type 16 is covered by the currently approved and available vaccines (Cervarix and Gardasil) [22,23] adopted for the Vaccine Alliance (Gavi)-supported vaccine roll-out programs in Nigeria which started in October 2023 [24]. This further highlights the observations of several other studies reporting a high frequency of non-vaccine HPV types among WLHIV in Sub-Saharan Africa [21], thus, the need to reinforce the implementation of population-based HR-HPV screening programs as a crucial step in preventing cervical cancer among WLHIV across the Sub-Saharan Africa region. The age-specific prevalence rates of HR-HPV infections among WLHIV, as depicted in our study reveal a distinct trend of decreasing prevalence with increasing age [25–27]. The high prevalence seen in women aged 25–39 years is consistent with the epidemiological understanding of HR-HPV infections, where younger women tend to have higher prevalence rates due to factors such as higher rates of sexual activity, new sexual partnerships, and potentially less robust immune responses compared to older women [28] while the second peak in the 50–54 years age group may be attributable to factors such as hormonal changes associated with menopause which affect vaginal and cervical epithelium, making it more susceptible to HPV infections [27]. Additionally, immune senescence, or the natural decline in immune function with age [29], may contribute to the reactivation or persistence of latent HR-HPV infections in older women.

Our study found no significant association between the baseline participant factors and HR-HPV infections, and this lack of association suggests that HR-HPV infections may be widespread across various demographic and behavioural profiles within the population of WLHIV. It also highlights the potential influence of the immunosuppressive state induced by HIV on the susceptibility to HR-HPV, overshadowing other sociodemographic and behavioural risk factors. However, our study identified that specific factors such as CD4+ cell count below 560 cells/μL and HPV 16 or 18 co-infection were associated with multiple HR-HPV infections. The finding of a significantly higher risk of HPV infections among HIV-infected women with a serum CD4+ cell count below 560 cells/μL is consistent with the findings from previous studies where a higher HR-HPV prevalence was recorded in HIV-infected women with lower CD4+ cell counts [12,30]. This is based on the understanding that a lower CD4+ cell count, indicative of more advanced immunosuppression, impairs the body’s ability to clear HPV infections, thereby increasing the likelihood of multiple HR-HPV infections [30]. This finding emphasizes the importance of maintaining optimal immune function through ART to mitigate the risk of HPV co-infections and their potential complications in WLHIV. Furthermore, the finding of an independent association between HPV 16 or 18 co-infection and multiple HR-HPV infections suggests a synergistic pathogenic mechanism that exacerbates the infection risk and the high oncogenic potential of HPV 16 and 18 responsible for a significant proportion of cervical cancers globally [1,31] which underscores the need for extended-spectrum genotype-specific monitoring and interventions.

The findings of our study have several important implications. Firstly, they highlight the critical need for regular and comprehensive HPV screening programs in addition to vaccinations among WLHIV. Secondly, the association between lower CD4+ cell counts, and multiple HR-HPV infections reinforces the importance of effective ART adherence to maintain immune function and reduce the burden of cervical cancer. However, the study has a few limitations. First, as the participating women were enrolled in the urban Lagos metropolis with the exclusion of those living in the slums and suburban areas of Lagos, the study findings can only be these settings in Lagos. Secondly, due to the cross-sectional nature of the study, we are unable to infer causality inference in the observed associations between the identified factors and multiple HR-HPV infections. Finally, as the study was conducted in an HIV treatment centre where all the enrolled women were on combined ART, our findings may not be entirely representative of HIV-infected women who are not taking treatment.

## Conclusions

Our study reveals that one in seven enrolled HIV-infected women had HR-HPV infections while one in 27 had multiple HR-HPV infections. None of the baseline participants’ factors was associated with HR-HPV infections. However, having a serum CD4+ cell count below 560 cells/μL and a co-infection with either HPV 16 or 18 genotype were significantly associated with the detection of multiple HR-HPV infections. These findings provide valuable insights into the epidemiology of HR-HPV infections and highlight the need for targeted screening, effective ART management, and vaccination programs to improve health outcomes and reduce the burden of cervical cancer in this vulnerable population.

## Figures and Tables

**Table 1:** Sociodemographic and clinical characteristics of the HIV-infected women participants (n=516)

Characteristics	Number (%)
**Mean age at enrolment (± SD) in years**	46.5 ± 7.3
**Mean BMI (± SD) in kg/m ^2^**	26.9 ± 15.3
**Mean CD4+ cell count (IQR) in cells/μL**	558.6 ± 172.2
**Mean age at menarche (± SD) in years**	15.2 ± 3.5
**Mean age at coitarche (± SD) in years**	19.9 ± 3.5
**Mean age at first pregnancy (± SD) in years**	23.4 ± 7.5
**Menstrual status**	
Premenopausal	308 (59.7)
Postmenopausal	208 (40.3)
**Parity**	
Nulliparous	26 (5.0)
Parous	490 (95.0)
**Previous vaginal birth**	
Yes	393 (76.2)
No	123 (23.8)
**Marital status**	
Single	49 (9.5)
Married	415 (80.4)
Separated	7 (1.4)
Divorced	9 (1.7)
Widowed	36 (7.0)
**Educational status**	
Uneducated	10 (1.9)
Primary education	58 (11.2)
Secondary education	269 (52.1)
Tertiary education	162 (31.4)
Postgraduate education	17 (3.3)
**Employment status**	
Unemployed	9 (1.7)
Housewife	16 (3.1)
Artisan	9 (1.7)
Trading	333 (64.5)
Privately/self employed	84 (16.3)
Civil servant	65 (12.6)
**Use of oral contraceptive**	
Yes	68 (13.2)
No	448 (86.8)
**Number of lifetime sexual partners**	
One	189 (36.6)
More than one	327 (63.4)
**Previous STI**	
Yes	106 (20.5)
No	410 (79.5)
**Alcohol consumption**	
Yes	99 (19.2)
No	417 (80.8)
**HR-HPV infections**	
Positive	69 (13.4)
Negative	447 (86.6)
**Multiple HR-HPV infections**	
Yes	19 (3.7)
No	497 (96.3)

Abbreviations: BMI, body mass index; HR-HPV, high-risk human papillomavirus; IQR, interquartile range; SD, standard deviation; STI, sexually transmitted infections.

a Values are given as mean ± SD, median (interquartile range), or frequency (percentage) unless indicated otherwise.

**Table 2:** Bivariable and multivariable analyses of factors associated with high-risk human papillomavirus infections

Factor	HR-HPV Infections		Bivariate *p* -value	OR (95% CI)	Multivariable *p* -value
	Positive (%)	Negative (%)			
**Number of participants**	69 (13.4)	447 (86.6)	NA	NA	NA
**Participants age**					
≥47 years	36 (14.3)	216 (85.7)	0.551	1.29 (0.75–2.21)	0.352
<47 years	33 (12.5)	231 (87.5)		1.00	Reference
**BMI**					
≥27 kg/m ^2^	40 (14.8)	231 (85.2)	0.330	NA	NA
<27 kg/m ^2^	29 (11.8)	216 (88.2)			
**Recent CD4+ cell count**					
≤560 cells/μL	40 (15.6)	217 (84.4)	0.145	1.33 (0.78–2.28)	0.300
>560 cells/μL	29 (11.2)	230 (88.8)		1.00	Reference
**Age at menarche**					
≥15 years	35 (12.5)	246 (87.5)	0.504	NA	NA
<15 years	34 (14.5)	201 (85.5)			
**Age at coitarche**					
<20 years	34 (14.7)	198 (85.3)	0.439	NA	NA
≥20 years	35 (12.3)	249 (87.7)			
**Age at first pregnancy**					
<23 years	37 (17.1)	180 (82.9)	0.036	1.64 (0.98–2.74)	0.060
≥23 years	32 (10.7)	267 (89.3)		1.00	Reference
**Menstrual status**					
Postmenopausal	31 (14.9)	177 (85.1)	0.430	NA	NA
Premenopausal	38 (12.3)	270 (87.7)			
**Parity**					
Parous	63 (12.9)	427 (87.1)	0.136	0.72 (0.26–1.98)	0.524
Nulliparous	6 (23.1)	20 (76.9)		1.00	Reference
**Previous vaginal birth**					
Yes	53 (13.5)	340 (86.5)	0.892	NA	NA
No	16 (13.0)	107 (87.0)			
**Marital status**					
Married	53 (12.8)	362 (87.2)	0.416	NA	NA
Unmarried	16 (15.8)	85 (84.2)			
**Educational status**					
Less than tertiary education	46 (13.6)	291 (86.4)	0.799	NA	NA
At least tertiary education	23 (12.8)	156 (87.2)			
**Employment status**					
Economically unengaged	7 (28.0)	18 (72.0)	0.028	2.43 (0.96–6.10)	0.060
Economically engaged	62 (12.6)	429 (87.4)		1.00	Reference
**Use of oral contraceptive**					
Yes	11 (16.2)	57 (83.8)	0.466	NA	NA
No	58 (12.9)	390 (87.1)			
**Lifetime sexual partners**					
More than one	48 (14.7)	279 (85.3)	0.251	NA	NA
One	21 (11.1)	168 (88.9)			
**Previous STI**					
Yes	19 (17.9)	87 (82.1)	0.122	1.52 (0.85–2.72)	0.161
No	50 (12.2)	360 (87.8)		1.00	Reference
**Alcohol consumption**					
Yes	13 (13.1)	86 (86.9)	0.938	NA	NA
No	56 (13.4)	361 (86.6)			

Abbreviations: BMI, body mass index; CI, confidence interval; HR-HPV, high-risk human papillomavirus; NA, not applicable; OR, adjusted odds ratio; STI, sexually transmitted infections.

**Table 3:** Bivariable and multivariable analyses of factors associated with multiple high-risk human papillomavirus infections

Variable	Multiple HR-HPV Infections		Bivariate *p* -value	OR (95% CI)	Multivariable *p* -value
	Yes (%)	No (%)			
**Number of participants**	19 (3.7)	497 (96.3)	NA	NA	NA
**Participants age**					
≥47 years	13 (5.2)	239 (94.8)	0.082	2.79 (0.91–8.54)	0.072
<47 years	6 (2.3)	258 (97.7)		1.00	Reference
**BMI**					
≥27 kg/m ^2^	10 (3.7)	261 (96.3)	0.992	NA	NA
<27 kg/m ^2^	9 (3.7)	236 (96.3)			
**Recent CD4+ cell count**					
≤560 cells/μL	13 (5.1)	244 (94.9)	0.098	3.32 (1.06–10.38)	0.039
>560 cells/μL	6 (2.3)	253 (97.7)		1.00	Reference
**Age at menarche**					
≥15 years	11 (3.9)	270 (96.1)	0.759	NA	NA
<15 years	8 (3.4)	227 (96.6)			
**Age at coitarche**					
<20 years	11 (4.7)	221 (95.3)	0.248	NA	NA
≥20 years	8 (2.8)	276 (97.2)			
**Age at first pregnancy**					
<23 years	10 (4.6)	207 (95.4)	0.341	NA	NA
≥23 years	9 (3.0)	267 (97.0)			
**Menstrual status**					
Postmenopausal	10 (4.8)	198 (95.2)	0.265	NA	NA
Premenopausal	9 (2.9)	299 (97.1)			
**Parity**					
Parous	18 (3.7)	472 (96.3)	0.964	NA	NA
Nulliparous	1 (3.8)	25 (96.2)			
**Previous vaginal birth**					
Yes	16 (4.1)	377 (95.9)	0.402	NA	NA
No	3 (2.4)	120 (97.6)			
**Marital status**					
Married	17 (4.1)	398 (95.9)	0.311	NA	NA
Unmarried	2 (2.0)	99 (98.0)			
**Educational status**					
Less than tertiary education	13 (3.9)	324 (96.1)	0.772	NA	NA
At least tertiary education	6 (3.4)	173 (96.6)			
**Employment status**					
Economically unengaged	2 (8.0)	23 (92.0)	0.240*	NA	NA
Economically engaged	17 (3.5)	474 (96.5)			
**Use of oral contraceptive**					
Yes	2 (2.9)	66 (97.1)	0.728	NA	NA
No	17 (3.8)	431 (96.2)			
**Lifetime sexual partners**					
More than one	13 (4.0)	314 (96.8)	0.642	NA	NA
One	6 (3.2)	183 (96.8)			
**Previous STI**					
Yes	5 (4.7)	101 (95.3)	0.526	NA	NA
No	14 (3.4)	396 (96.6)			
**Alcohol consumption**					
Yes	6 (6.1)	93 (93.9)	0.162	2.06 (0.65–10.38)	0.221
No	513 (3.1)	404 (96.9)		1.00	Reference
**HPV 16/18 genotype**					
Yes	8 (47.1)	9 (52.9)	<0.001	38.98 (11.93–127.37)	<0.001
No	11 (2.2)	488 (97.8)		1.00	Reference

Abbreviations: BMI, body mass index; CI, confidence interval; HR-HPV, high-risk human papillomavirus; NA, not applicable; OR, adjusted odds ratio; STI, sexually transmitted infections.

## Data Availability

The datasets used and/or analyzed during the current study are available from the corresponding author (KSO) upon reasonable request.
